# Calvarial bone grafts to augment the alveolar process in partially dentate patients: a prospective case series

**DOI:** 10.1186/s40729-020-00251-5

**Published:** 2020-09-24

**Authors:** Ahmed Yousif, Gerry M. Raghoebar, Thomas F. Putters, Arjan Vissink, Jurjen Schortinghuis

**Affiliations:** 1 Department of Oral and Maxillofacial Surgery, University Hospital Groningen, University Medical Center Groningen, Groningen, The Netherlands; 2 Department of Oral and Maxillofacial Surgery, Treant Scheper Hospital, Emmen, The Netherlands

## Abstract

**Background:**

Calvarial bone grafts as a pre-implant augmentation procedure are mostly used to reconstruct the edentulous maxilla, although calvarial grafts could also be used in the partially dentate patients needing extensive bone grafting.

**Methods:**

In 7 consecutive partially dentate patients needing bone grafting because of a large bony defect as a result of trauma (*n* = 1), oligodontia (*n* = 1), failed previous bone augmentation (*n* = 1), or atrophy (*n* = 4), the alveolar process was reconstructed with calvarial bone as a pre-implant procedure.

**Results:**

A total of 30 implants was placed either immediate at the time of bone grafting (13 implants) or after a healing time of 4 months when immediate placement was not possible (17 implants). One wound dehiscence occurred that needed secondary intervention. During follow-up (40 ± 14 months), one implant was lost due to peri-implantitis with an infected osteosynthesis screw. Marginal peri-implant bone loss was 0.65 ± 0.47 mm during this period.

**Conclusion:**

Calvarial bone is a sound extra-oral donor site when aiming for reconstruction of a large bony defect of the alveolar process of partially dentate patients.

## Introduction

When teeth are lost, the remaining defect in the dental arch can be bothersome to the patient for aesthetic or functional reasons. A way to restore the dental arch is the placement of dental implant-supported prosthodontics. In case of an insufficient bone volume at the defect location, a reconstructive procedure is needed to allow for a sufficient bone volume to support the implants, e.g., a local bone augmentation [1].

When there is substantial loss of bone not allowing for reliable placement of the implants, a bone augmentation is needed. For large bone defects, extra-oral donor sites may be needed. The most common extra-oral grafting site is still the anterior iliac crest [2]. Although copious amounts of bone can be harvested from this site, major disadvantages of harvesting bone from the anterior iliac crest are donor site morbidity (pain and gait problems) and the unpredictable resorption of the graft after grafting [3]. Calvarial bone grafts can serve as an alternative to anterior iliac crest bone grafts. Major advantages of harvesting calvarial bone is the lower early donor site morbidity [4, 5] and less resorption of the bone graft during follow-up [6]. A limitation is possible scar visibility in bald patients or palpable contour deficits [5].

In this prospective cases series, we report the outcome of calvarial bone grafting in 7 partially dentate patients needing a pre-implant augmentation procedure with extra-oral bone.

## Case series

Between 2012 and 2018, a reconstruction of a large bone defect was performed with extra-oral bone to allow for implant placement was performed in seven consecutive partially edentate patients (Table 1). A large bone defect was defined as less than 2 mm in width of the alveolar process and in vertical direction in more than 4 mm. The patients did not use bisphosphonates and immunosuppressive medications. As large amounts of bone were needed, it was decided to harvest calvarial bone grafts using the technique described by Schortinghuis et al. [7] and Putters et al. [8]. Perioperative complications such as dura exposure, dura leakage, and graft fracture were noted.

**Table 1:** Partially dentate patients in whom the alveolar process was reconstructed with calvarial bone

No.	Gender, age (years)	Cause	Location	Number of implants	Immediate or delayed placement, brand implant (B = Biomet, S= Straumann)	Follow-up (months); complications
1	F, 35	Trauma horsekick to face	Premaxilla	3	Immediate, B	58; 1 implant lost due to infected osteosynthesis screw/peri-implantitis; some hair loss along scar.
2	F, 43	Oligodontia	Premaxilla, symphysis	7	Delayed, S	21; some hair loss along scar
3	M, 36	Failed previous bone augmentation	Premaxilla	1	Delayed, S	28; wound dehiscence
4	F, 68	Atrophy	Lateral maxilla and mandibe both sides	7	Immediate, S	47; no complications
5	M, 55	Atrophy	Premaxilla	3	Immediate, S	53; no complications
6	F, 65	Atrophy	Lateral mandible both sides	4	Delayed, S	34; no complications
7	F, 69	Atrophy	Lateral maxilla	5	Delayed, B	38; no complications

*F* female, *M* male, *B* Biomet Nanotite implants (ZimmerBiomet, Dordrecht, The Netherlands), *S* Straumann bone level implants (Straumann, Wolhusen, Switserland)

First, the alveolar process was augmented with calvarial bone. When there was enough space between the osteosynthesis screws, the dental implants were placed immediately. After 4 months, the implants were retrieved, healing abutments placed, and screws that were palpable were removed. When after the augmentation there was not enough space between the screws to immediately place the implants, the implants were placed after 4 months. All screws were removed before implant placement. Postoperatively, pain (Visual Analogue Scale; VAS), wound dehiscence extra-/intra-oral, hair loss, peri-implant bone loss and implant loss were recorded.

Peri-implant bone height levels were measured during follow-up on calibrated orthopanthomographs with laser guidance positioning (Planmeca Promax, Helsinki, Finland) taken directly postoperatively, after 6 weeks, 6 months and 1 year after implant placement as well as yearly after placement of suprastructure. The average peri-implant marginal bone loss on the yearly made calibrated orthopantomograms was assessed by the independent researcher (AY).

Patient satisfaction was assessed 6 months after functional loading of the suprastructures using questionnaires where the patients would rate their answer on a 5-point scale (0 = very dissatisfied; 5 = very satisfied). The questions were whether they were satisfied with their ability to chew, with the aesthetic appearance of the end result, whether they would recommend the procedure to others and whether they would undergo the same procedure again. Also an overall satisfaction rate of the treatment was asked (1 = worst outcome; 10 = best outcome).

## Results

**Fig. 1:**
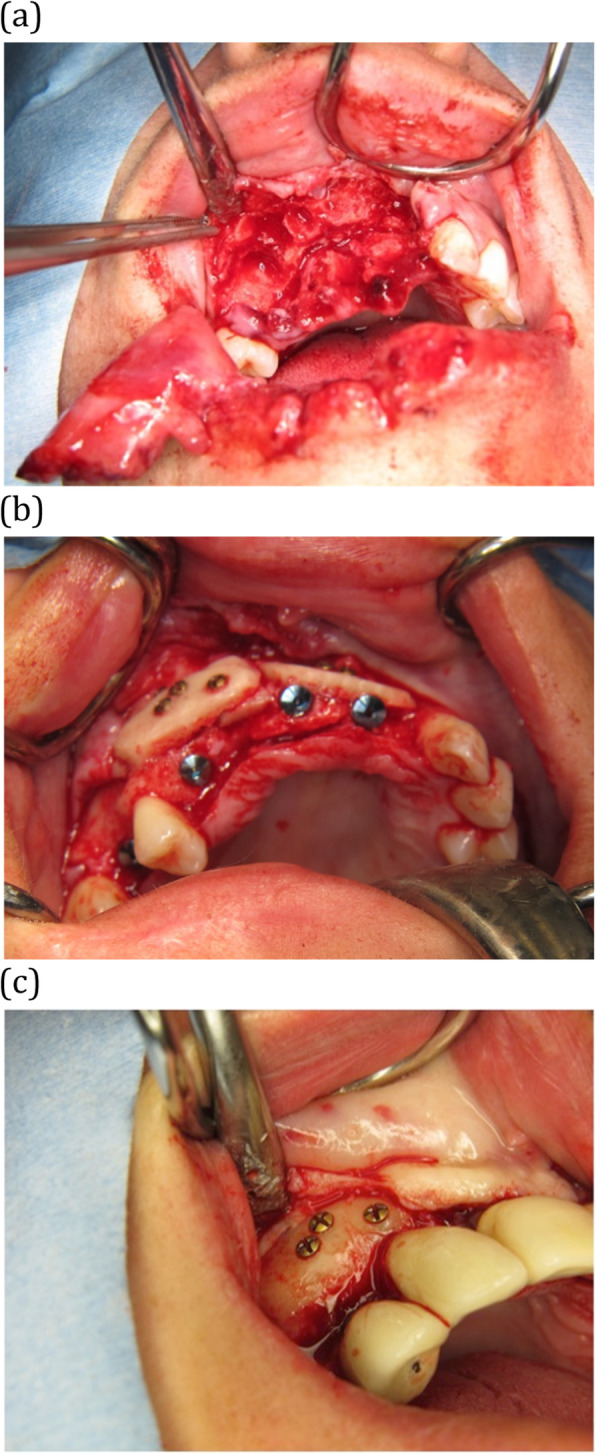
A 35-year-old woman with a traumatic bony defect region 11–14 due to a horse kick. **a** Clinical image of the traumatic avulsion of teeth and maxillary bone tooth locations 11–14 and partial avulsion of the lower lip. **b** Intraoperative photograph showing immediate implant placement after calvarial bone grafting to the maxilla. Using a prefabricated drill guide, the calvarial bone blocks were placed and fixed with microscrews in such a way that the implants could be placed between them in the prosthodontically required position. **c** After 4.5 years, the implant at location 11 had to be removed due to an infected osteosynthesis screw and peri-implantitis. The photograph of the grafted locations 13–14 showed that the screw heads were still in close contact with the previously grafted area. This observation underlines that there is minimal surface resorption of the calvarial bone almost 4.5 years of functional loading

**Fig. 2:**
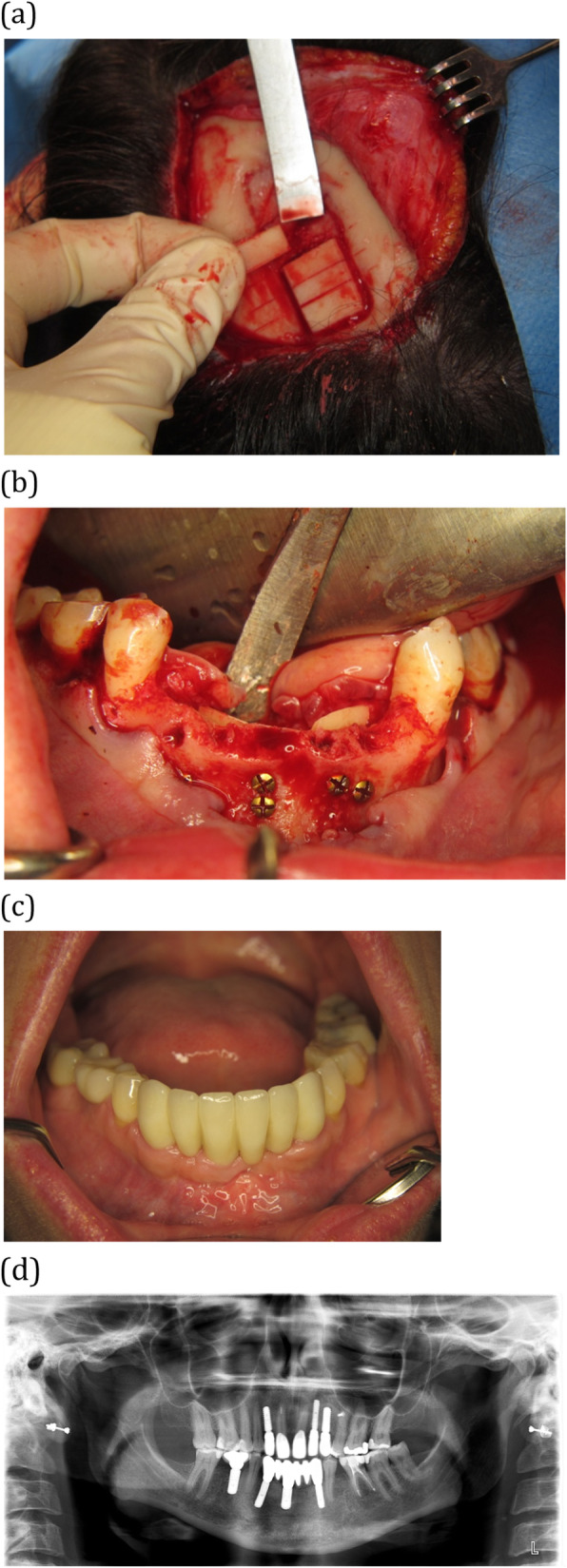
A 43-year-old female with oligodontia with missing tooth numbers 12, 22, 23, 35, 33–43 and 45. **a** Harvesting of outer table calvarial bone blocks. The blocks were harvested piece-by-piece. **b** Reconstruction of the mandible at tooth locations 33–43 by placing the calvarial grafts lingually. The grafts were fixed with screws from the buccal side. The implants were placed after 4 months. **c** Clinical image of the end result. A fixed bridge was made in the region 33–43. **d** Orthopantomogram of the end result at 21 months follow-up showing negligible marginal bone loss around the implants

Six females and one male patient, aged 53 ± 15 years at the time of treatment, received calvarial bone grafting of large bony defects of the maxilla or mandible (Table 1). These defects were the result of trauma (*n* = 1, Fig. 1), oligodontia (*n* = 1, Fig. 2) failed previous bone augmentation (*n* = 1) or atrophy (*n* = 4). A total of 30 implants was placed of which 13 implants immediate at the time of the calvarial grafting and 17 implants in augmented bone after 4 months of healing.

The choice to immediately place implants at the time of calvarial grafting or to delay their placement after 4 months of healing of the calvarial grafts was based on the anatomy of the remaining alveolar process and the position of the osteosynthesis screws. In case of a knife edge ridge, a calvarial graft can be fixed with screws on either side, and this allows an implant to be placed between the screws. In case there is not enough distance between the screws when the graft is too small as in the case of a single tooth augmentation, the implant placement is performed after 4 months.

From these patients in whom the implants were placed after bone healing, it became obvious that resorption of the grafted sites was negligible as the screw heads of the screws that were used to fixate the calvarial bone were still in contact with the surface of the graft. After removal of the screws, the implant bed could be drilled with ease according to the planned position using a surgical guide.

Average postoperative VAS score was 3.5 ± 2.0 the first postoperative day and 3.0 ± 1.5 after 7 days, decreasing to zero within 14 days. The surgical procedures were uneventful. No perioperative complications occurred. Postoperatively, one persistent wound dehiscence occurred in a patient where a substantial vertical and horizontal bone defect at tooth location 11 was reconstructed with a buccal and palatal calvarial graft (double plating technique). This was successfully managed with additional surgical wound closure and hyperbaric oxygen therapy [9]. Mean follow-up was 40 ± 14 months. During follow-up, two patients mentioned minimal hair loss at the location of the scar on the scalp. One implant was lost after 4.5 years due to infection of an exposed osteosynthesis screw and peri-implantitis.

Mean peri-implant marginal bone loss after 1 year of functional loading was 0.48 ± 0.58 mm that increased to 0.65 ± 0.47 mm during the total follow-up of 40 months. All patients were very satisfied with their ability to chew (all scored 5). Four patients were very satisfied (score 5) and three patients satisfied (score 4) with the aesthetic appearance. All patients would recommend the procedure to others and would undergo the same procedure again. The overall satisfaction score of the patients was 9.2 ± 0.4.

## Discussion

In this study, we present an application for the use of calvarial bone grafts: to reconstruct the partially dentate alveolar process when a substantial amount of bone is needed as a pre implant procedure. The technique of immediate implant placement after augmentation with calvarial bone has been previously described with high success, but this concerned the edentulous maxilla and not the maxilla and mandible of partially dentate patients [6].

Perioperatively, no complications occurred. This is in line with other studies that indicate the complication rate of harvesting calvarial bone is negligible, especially using a safe technique [8]. Perioperatively, the calvarial bone could be handled well, and the pieces fitted nicely onto the alveolar process.

One implant was lost (out of 30) in our series in a patient who had traumatic bone loss in the maxilla after a horse kick to the face (Fig. 1). In this patient, the implants were retrieved, and healing abutments were placed after 4 months without removing the osteosynthesis screws because they were not palpable. After 4.5 years, however, one more cranially placed screw became infected possibly due to a localized peri-implantitis and led to bone and implant loss at tooth location 11. At re-entry, it was observed that the calvarial bone graft on the healthy side had not resorbed as the screw heads were still in contact with the surface of the calvarial bone graft. This illustrates that calvarial bone grafts do not tend to resorb over quite a long time (years). Based on this case, we now routinely remove the screws that are in close vicinity to the oral cavity when implants are retrieved.

Average loss of peri-implant bone height was < 1 mm during follow-up (mean 40 months). This is considered a high success rate (100%) according to the criteria of Albrektsson [10] and supports our hypothesis that calvarial bone augmentation is a promising technique with favourable long-term results.

Two patients mentioned minor hair loss at the scar location. This was probably due to the use of diathermy to coagulate bleeding vessels of the scalp, also coagulating hair follicles. The overall patient satisfaction score was nevertheless high, and patients would undergo the treatment again if needed. We modified our technique to use diathermy more sparcely.

Calvarial bone can be harvested safely and has been shown to be accompanied by minor morbidity with low direct postoperative pain levels. The patient-reported outcome measures confirmed that bone graft harvesting from the calvarium is an appropriate procedure, reflected by high levels of satisfaction, minor long-term sequela and improvement of perceived oral health [5]. A limitation of calvarial bone harvesting may be visible scarring in the bald patient, and possible contour deficits afterwards [5].

To our knowledge, there are two case series published in which calvarial bone grafts are used to reconstruct the partially dentate alveolar process [11, 12]. Lozano et al. [11] reconstructed large maxillary horizontal and vertical defects in partially dentate patients with calvarial bone grafts. A total of 10 patients were reconstructed. Twenty-two implants were placed after a minimal healing time of 15 weeks. Vertical bone loss was 0.78 mm after 41 months after implantation. These results are in accordance with those of our study (0.65 mm after 40 months). Monje et al. [12] reconstructed maxillary defects with either calvarial bone or with mandibular ramus bone in 10 partially dentate patients [12]. They compared bone microstructure and the primary stability of the implants placed after 4–6 months in either calvarial or ramus bone by measuring resonance frequency and histomorphometric and micro-CT analysis of bone biopsies. This study focused on bone quality and not on implant survival and bone loss. Both calvarian and ramus block grafts behave similarly with regard to their bone-related morphometric parameters, and there was no difference in primary implant stability.

Other studies reported in the literature on calvarial grafting as pre-implant procedure mostly focus on the fully edentulous severely resorbed maxilla [13, 14] or the edentulous mandible [15].

It is important to consider the amounts of bone required for the reconstruction of a large bone defect when selecting a donor site. When only a limited amount of autologous bone is needed in partially dentate cases, intra-oral bone grafting sites can be used such as retromolar [16], maxillary tuberosity or chin area [17] with or without the use of bone substitutes and/or barrier membranes. Although intra-oral bone grafting prevents the need for an extra-oral donor site with associated morbidity, for larger defects, the calvarial bone seems suitable. This is illustrated by our case in which two attempts with right and later left retromolar bone to reconstruct a defect at tooth location 11 had failed, and there was need for a horizontal and vertical reconstruction. Here, calvarial bone was considered ideal for its compact structure, easy handling and minimal resorption.

## Conclusion

Partially dentate cases needing extensive bone grafting are quite uncommon in general practice. Our case series illustrate that calvarial bone grafts can be considered as a pre-implant augmentation procedure in cases where a partially dentate alveolar process needs to be augmented with a substantial amount of bone.

## Data Availability

The datasets used and/or analysed during the current study are available from the corresponding author on reasonable request.

## Abbreviations

VAS: Visual Analogue Scale
